# Blimp-1 Rather Than Hobit Drives the Formation of Tissue-Resident Memory CD8^+^ T Cells in the Lungs

**DOI:** 10.3389/fimmu.2019.00400

**Published:** 2019-03-07

**Authors:** Felix M. Behr, Natasja A. M. Kragten, Thomas H. Wesselink, Benjamin Nota, Rene A. W. van Lier, Derk Amsen, Regina Stark, Pleun Hombrink, Klaas P. J. M. van Gisbergen

**Affiliations:** ^1^Department of Hematopoiesis, Sanquin Research and Landsteiner Laboratory, Amsterdam UMC, University of Amsterdam, Amsterdam, Netherlands; ^2^Department of Experimental Immunology, Amsterdam UMC, University of Amsterdam, Amsterdam, Netherlands; ^3^Department of Molecular and Cellular Hemostasis, Sanquin Research and Landsteiner Laboratory, Amsterdam UMC, University of Amsterdam, Amsterdam, Netherlands

**Keywords:** hobit, blimp-1/PRDM1, lung T cell, T cell differentiation, influenza virus infection, central memory CD8(+) T cells, tissue-resident memory CD8(+) T cells, TCF-1

## Abstract

Tissue-resident memory CD8^+^ T (T_RM_) cells that develop in the epithelia at portals of pathogen entry are important for improved protection against re-infection. CD8^+^ T_RM_ cells within the skin and the small intestine are long-lived and maintained independently of circulating memory CD8^+^ T cells. In contrast to CD8^+^ T_RM_ cells at these sites, CD8^+^ T_RM_ cells that arise after influenza virus infection within the lungs display high turnover and require constant recruitment from the circulating memory pool for long-term persistence. The distinct characteristics of CD8^+^ T_RM_ cell maintenance within the lungs may suggest a unique program of transcriptional regulation of influenza-specific CD8^+^ T_RM_ cells. We have previously demonstrated that the transcription factors Hobit and Blimp-1 are essential for the formation of CD8^+^ T_RM_ cells across several tissues, including skin, liver, kidneys, and the small intestine. Here, we addressed the roles of Hobit and Blimp-1 in CD8^+^ T_RM_ cell differentiation in the lungs after influenza infection using mice deficient for these transcription factors. Hobit was not required for the formation of influenza-specific CD8^+^ T_RM_ cells in the lungs. In contrast, Blimp-1 was essential for the differentiation of lung CD8^+^ T_RM_ cells and inhibited the differentiation of central memory CD8^+^ T (T_CM_) cells. We conclude that Blimp-1 rather than Hobit mediates the formation of CD8^+^ T_RM_ cells in the lungs, potentially through control of the lineage choice between T_CM_ and T_RM_ cells during the differentiation of influenza-specific CD8^+^ T cells.

## Introduction

Long-term memory of previously encountered pathogens is crucial to enable enhanced responses of our body's immune defenses in future encounters with the same pathogen. Antigen-specific memory CD8^+^ T cells form an essential aspect of immunological memory. Particularly in the context of local infections, tissue-resident memory CD8^+^ T (T_RM_) cells situated in the originally infected tissues are important for protective immunity (1). Their position in barrier tissues, including skin, intestine, female reproductive tract and lungs, places non-circulating CD8^+^ T_RM_ cells at sites of pathogen entry, where they provide efficient early protection against local reinfection (2–5). The superior protective capacity of CD8^+^ T_RM_ cells is mediated through direct effector functions and by promoting the activation and recruitment of other immune cells. Upon antigen encounter, CD8^+^ T_RM_ cells rapidly release pro-inflammatory cytokines, including interferon-γ (IFN-γ), which induce a tissue-wide state of alert and initiate the recruitment of circulating B cells and memory T cells (4, 6, 7). In some tissues, CD8^+^ T_RM_ cells can also directly lyse target cells and limit pathogen spread by employing cytotoxic mechanisms (8, 9). Given their potency in limiting pathogen spread, insights into the mechanisms regulating the development and maintenance of CD8^+^ T_RM_ cells may contribute to improved strategies to induce protective immunity via vaccination (10, 11).

The localization of CD8^+^ T_RM_ cells to different organs suggests the existence of tissue-specific adaptions, which, in turn, may influence the local development and maintenance of CD8^+^ T_RM_ cells. Compared to CD8^+^ T_RM_ cells in the skin and intestine, lung CD8^+^ T_RM_ cells are distinct at the transcriptional level (12), indicating specific adaptions to the local microenvironments. Lung CD8^+^ T_RM_ cells share common features with CD8^+^ T_RM_ cells from other tissues, including the expression of tissue-retention molecules such as CD69 and the α_E_ integrin CD103 (13, 14). Importantly, lung CD8^+^ T_RM_ cells provide robust protection against heterosubtypic influenza virus infection (5, 15). However, CD8^+^ T_RM_ cells in the lungs are divergent from CD8^+^ T_RM_ cells at other peripheral sites in terms of their maintenance. While CD8^+^ T_RM_ cells in most tissues are long-lived and self-sustaining (2, 16), the virus-specific CD8^+^ T_RM_ cell population in the lungs declines over time after pulmonary infection, which coincides with waning of heterosubtypic immunity to influenza virus (5, 14, 17). The mechanisms underlying this limited longevity of lung CD8^+^ T_RM_ cells are not fully understood. After pulmonary infection, CD8^+^ T_RM_ cells localize to specific niches at sites of tissue regeneration in the lung, and it has been suggested that the disappearance of these niches over time may account for the limited longevity of lung CD8^+^ T_RM_ cells (18). The preservation of the resident population in the lungs may additionally require continuous replenishment from a circulating effector memory CD8^+^ T (T_EM_) cell pool (17, 19). The gradual decline in the capacity of circulating memory CD8^+^ T cells to form CD8^+^ T_RM_ cells may thus contribute to the demise of the lung CD8^+^ T_RM_ cell population (17). Consequently, tissue residency might be differentially regulated at the transcriptional level for CD8^+^ T cells in the lung compared to other organs.

We have recently found that the transcription factor Hobit and its homolog Blimp-1 control the generation and/or maintenance of CD8^+^ T_RM_ cells across several tissues, including skin, small intestine, liver, and kidney (20). These transcription factors instruct a universal program of tissue-residency, in part by directly suppressing the tissue egress receptors CCR7 and S1PR1 (20). Here, we investigated the role of Hobit and Blimp-1 in the development of lung CD8^+^ T_RM_ cells after pulmonary influenza virus infection. CD8^+^ T_RM_ cells in the lungs exhibited high expression of Hobit and Blimp-1 at the transcript level. However, we found that Blimp-1, but not Hobit, is essential for the formation of lung CD8^+^ T_RM_ cells. Blimp-1 also limited the formation of central memory CD8^+^ T (T_CM_) cells. These findings highlight the unique transcriptional regulation of CD8^+^ T_RM_ cells in the lung, which may have implications for future influenza vaccination strategies.

## Results

### Lung CD8^+^ T_RM_ Cells Arising After Respiratory Influenza Virus Infection Express Hobit

**Figure 1:**
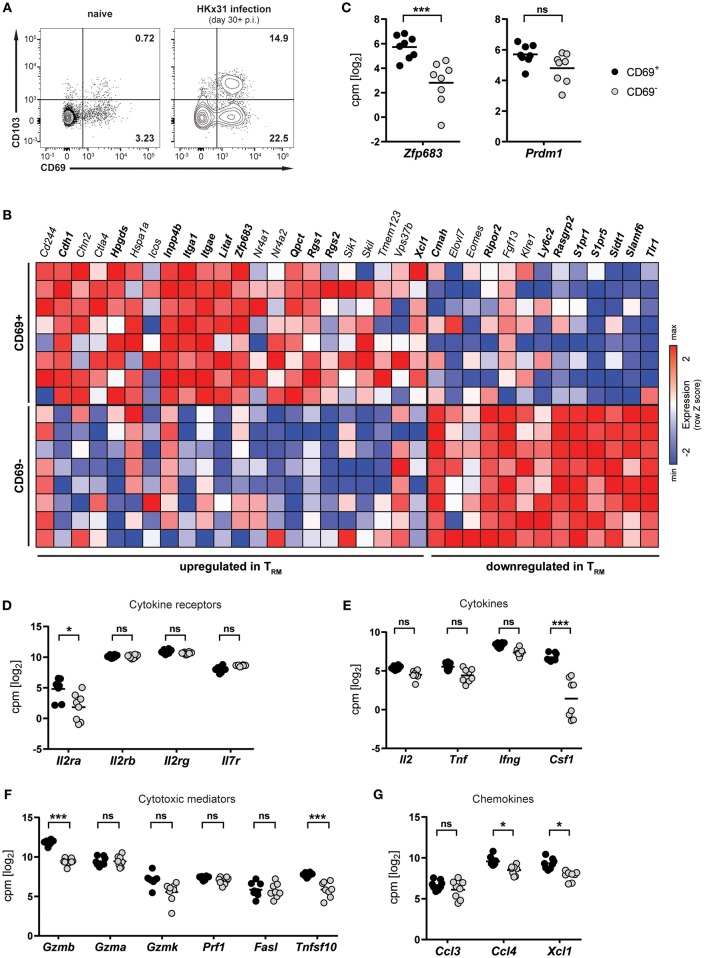
Transcriptional profile of lung CD8^+^ T_RM_ cells arising after influenza virus infection. **(A)** Representative flow cytometry plots are shown of CD69 and CD103 expression on memory (CD44^high^ CD62L^lo^) CD8^+^ T cells isolated from lungs of naïve mice or lungs of mice at day 30+ (memory phase) after intranasal HKx31 influenza virus infection. **(B)** Expression (column Z-score) of mRNA from genes belonging to the T_RM_ core signature (12) in CD69^+^ and CD69^−^ memory (CD44^high^ CD62L^lo^) CD8^+^ T cells isolated from murine lungs at day 30+ after HKx31 infection. Genes in bold are differentially expressed in CD69^+^ vs. CD69^−^ cells. Expression of gene *Usp33*, part of the T_RM_ core signature, was not detected by our analysis. **(C**–**G)** Expression (log2 read counts per million, voom normalized expression) of **(C)** Hobit (*Zfp683*) and Blimp-1 (*Prdm1*), genes encoding for **(D)** common γ-chain cytokine receptors, **(E)** cytokines, **(F)** cytotoxic mediators, and **(G)** selected chemokines is shown in CD69^+^ and CD69^−^ memory (CD44^high^ CD62L^lo^) CD8^+^ T cells isolated from murine lungs at day 30+ after HKx31 infection. Symbols represent individual mice and line indicates the mean. Data from one experiment (*n* = 8), taken from Hombrink et al. (23). ^*^FDR adjusted *P* < 0.05; ^***^ FDR adjusted *P* < 0.001; ns: not significant.

Infection of mice with influenza virus induces differentiation of virus-specific CD8^+^ T cells into CD8^+^ T_RM_ cells, which persist in the lung and provide protection against subsequent reinfection (5, 21, 22). To investigate lung CD8^+^ T_RM_ cells arising after influenza virus infection, mice were infected intranasally with HKx31 influenza A virus and CD8^+^ T cells were isolated and analyzed in the memory phase (day 30+ p.i.). Influenza virus infection gave rise to a substantial CD69^+^ CD8^+^ T_RM_ cell population in the lung, which partially expressed CD103 (Figure 1A). This population was nearly absent in lungs from naïve mice, indicating that the vast majority of CD8^+^ T_RM_ cells were a direct result of influenza virus infection. In mice, a core signature of gene-expression has been determined in CD8^+^ T_RM_ cells (12). Transcriptional profiling of CD69^+^ and CD69^−^ memory CD8^+^ T cells isolated from lungs of HKx31-immune mice (day 30+ p.i.) by RNA sequencing confirmed the resident phenotype of the CD69^+^ CD8^+^ T cell population arising after influenza virus infection (Figure 1B). When compared to the T_RM_ core signature, the obtained transcriptional profiles of lung CD8^+^ T_RM_ cells showed a good congruency. Genes associated with tissue-residency, including *Cdh1, Itga1, Itgae, Rgs1*, and *Rgs2*, were specifically upregulated in the CD69^+^ population, while genes associated with circulating memory CD8^+^ T cells, including tissue-egress factors (e.g., *S1pr1* and *S1pr5*), were substantially downregulated in these cells (Figure 1B). Overall, out of 35 genes of the T_RM_ core signature, 20 were significantly up- or downregulated in the CD69^+^ CD8^+^ T cell compartment compared to the CD69^−^ CD8^+^ T cell pool from the lung. Importantly, the transcription factor Hobit (encoded by *Zfp683*), which we have recently identified as a key regulator of tissue-residency (20), was also significantly upregulated in lung CD8^+^ T_RM_ cells, as compared to the circulating memory CD8^+^ T cell population in the lung (Figures 1B,C). In contrast, expression levels of the related transcription factor Blimp-1 (encoded by *Prdm1*) were not significantly different between the two memory subsets (Figure 1C). Members of the common γ-chain cytokines, in particular IL-2, IL-7, and IL-15, play an important role in the maintenance of memory CD8^+^ T cells. In the lung, both circulating and resident memory CD8^+^ T cells expressed the individual components of the IL-7 (*Il7r, Il2rg*) and IL-15 receptor (*l2rb, Il2rg*) (Figure 1D). In contrast, the alpha chain of the IL-2 receptor was upregulated in lung CD8^+^ T_RM_ cells compared to CD69^−^ memory CD8^+^ T cells in the lung. Furthermore, in comparison to their circulating counterparts in the lung, the CD69^+^ lung T_RM_ cells expressed significantly higher levels of pro-inflammatory cytokines, chemokines and cytotoxic mediators, including colony-stimulating factor-1 (*Csf1*), lymphotactin (*Xcl1*) granzyme B (*Gzmb*) and TNF-related apoptosis-inducing ligand (TRAIL, *Tnfsf10*), indicative of a poised effector state (Figures 1E–G). Taken together, influenza virus infection induced a distinct population of CD69^+^ CD8^+^ T cells in the lungs, which were identified as bona fide T_RM_ cells by transcriptional analysis. Importantly, these lung-resident CD8^+^ T cells exhibited elevated transcript levels of effector molecules and were characterized by high expression of the T_RM_-associated transcription factor Hobit.

### CD8^+^ T_RM_ Cell Formation in the Lung Requires Hobit and/or Blimp-1

**Figure 2:**
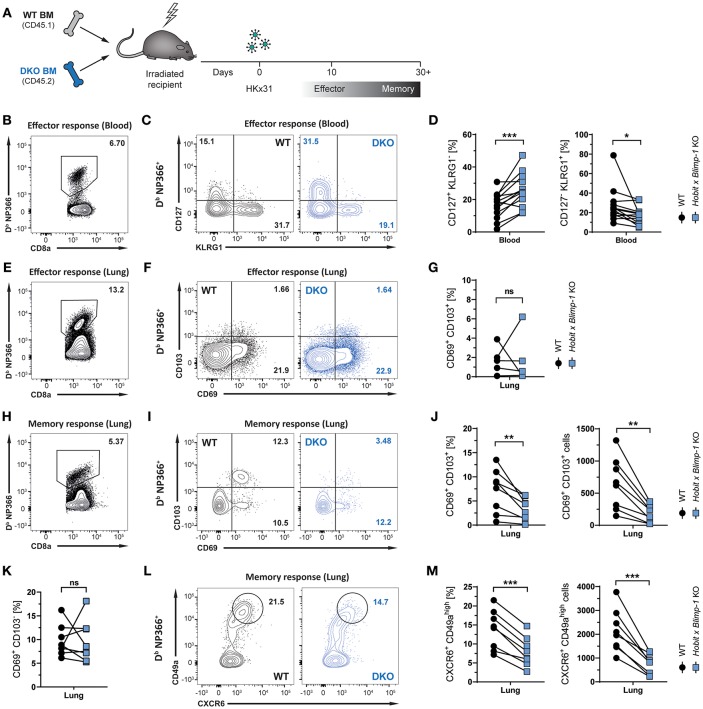
Formation of lung CD8^+^ T_RM_ cells depends on Hobit and/or Blimp-1. **(A)** Experimental scheme shows the generation of mixed bone marrow (BM) chimeras from WT and Hobit and Blimp-1 KO (DKO) mice (1:1 ratio) and HKx31 influenza virus infection of these chimeric mice. **(B–G)** Analysis at the effector time point is shown. **(B**,**E)** Representative flow cytometry plot shows frequency of D^b^ NP366^+^ cells within CD8^+^ T cell population in **(B)** blood and **(E)** lung at day 10 post infection. **(C**,**F)** Representative flow cytometry plots show **(C)** expression of CD127 and KLRG1 and **(F)** expression of CD69 and CD103 on D^b^ NP366^+^ donor CD8^+^ T cells of the WT and DKO compartment from **(C)** blood and **(F)** lungs at day 10 post infection. **(D**,**G)** The frequencies of **(D)** CD127^+^KLRG1^−^ and KLRG1^+^CD127^−^ and **(G)** CD69^+^ CD103^+^ D^b^ NP366^+^ donor CD8^+^ T cells of the WT and DKO compartment from **(D)** blood and **(G)** lungs at day 10 post infection were quantified. **(H–M)** Analysis at the memory time point is shown. **(H)** Representative flow cytometry plot shows frequency of D^b^ NP366^+^ cells within CD8^+^ T cell population in lung at day 30+ post infection. **(I**,**L)** Representative flow cytometry plots show **(I)** expression of CD69 and CD103 and **(L)** expression of CXCR6 and CD49a on D^b^ NP366^+^ donor CD8^+^ T cells of the WT and DKO compartment from lungs at day 30+ post infection. **(J**,**K**,**M)** The frequencies and absolute numbers of **(J)** CD69^+^CD103^+^, **(K)** CD69^+^CD103^−^, and **(M)** CXCR6^+^CD49a^high^ D^b^ NP366^+^ donor CD8^+^ T cells of the WT and DKO compartment from lungs at day 30+ post infection were quantified. Data from **(G)** one experiment (*n* = 5) or combined data from **(C**,**J**,**K**,**M)** two independent experiments (*n* = 8). Symbols represent individual mice, lines connect paired samples. Paired *t*-test. ^*^P < 0.05; ^**^P < 0.01; ^***^P < 0.001, ns: not significant.

Given its selective expression in lung CD8^+^ T_RM_ cells, we hypothesized that Hobit may contribute to the development of these cells. In other tissues, including the skin, liver, kidney, and small intestine, Hobit regulates the generation and/or maintenance of CD8^+^ T_RM_ cells together with its homolog Blimp-1 (20). In order to investigate the role of these two transcription factors in the development of lung CD8^+^ T_RM_ cells, mixed bone marrow (BM) chimeric mice were generated, containing a WT compartment and a compartment lacking functional Hobit and Blimp-1 (double knock-out, DKO) (Figure 2A). An approach with mixed BM chimeric mice was chosen to minimize indirect effects on CD8 T cell differentiation through differences in viral clearance. Mice were infected intranasally with HKx31 virus, and the virus-specific (D^b^ NP366^+^) CD8^+^ T cell response was analyzed over time. Previous studies have demonstrated a critical role for Blimp-1 in terminal effector cell (TEC) differentiation (24, 25). In line with these findings, analysis of virus-specific D^b^ NP366^+^ CD8^+^ T cells in the blood at the peak of the anti-viral effector CD8^+^ T cell response (day 10 p.i.) revealed a substantial decrease in KLRG1^+^ CD127^−^ TECs in the DKO compared to the WT compartment (Figures 2B–D). Concomitantly, D^b^ NP366^+^ cells deficient for both Hobit and Blimp-1 exhibited a sharp increase in CD127^+^ KLRG1^−^ memory precursor effector cells (MPECs) compared to their WT counterparts (Figures 2C,D). In lung tissue, a distinct CD69^+^ population was already observed at the effector stage, while CD103 expression was minimal (Figure 2F). Both the WT and the DKO compartment gave rise to similar frequencies of CD69^+^ CD103^−^ and CD69^+^ CD103^+^ cells at this stage, suggesting little impact of Hobit and Blimp-1 deficiency on the formation of these cells (Figures 2E–G). In contrast, D^b^ NP366^+^ DKO cells generated less T_RM_ cells in the lung at the memory phase than their WT counterparts (Figures 2H,I). This defect was most pronounced for CD69^+^ CD103^+^ cells, which were decreased in both frequencies and absolute numbers in the DKO compartment compared to the WT compartment (Figures 2I,J). Interestingly, DKO cells formed CD69^+^ CD103^−^ T_RM_ cells at near similar frequencies as WT cells, indicating little effect of combined Hobit and Blimp-1 deficiency on the generation of this population (Figures 2I,K). Apart from CD69 and CD103, CD8^+^ T_RM_ cells across tissues express additional tissue-residency markers, including the chemokine receptor CXCR6 and the integrin CD49a (26–29). Influenza-virus-specific WT CD8^+^ T cells in the lungs co-expressed CXCR6 and CD49a at similar frequencies as the residency marker CD69, suggesting that both molecules also identify CD8^+^ T_RM_ cells in this tissue (Figures 2L,M). Interestingly, combined deficiency for Hobit and Blimp-1 impaired the formation of CXCR6^+^ CD49a^high^ cells, which were decreased in both frequencies and absolute numbers in the DKO compartment compared to the WT compartment (Figures 2L,M). In all, these results show that the combined genetic ablation of Hobit and Blimp-1 results in reduced TEC and enhanced MPEC formation during the effector CD8^+^ T cell response, and impairs the generation of CD103^+^ lung T_RM_ cells in the memory CD8^+^ T cell response.

### Hobit and Blimp-1 Impair Formation of CD8^+^ T_CM_ Cells

**Figure 3:**
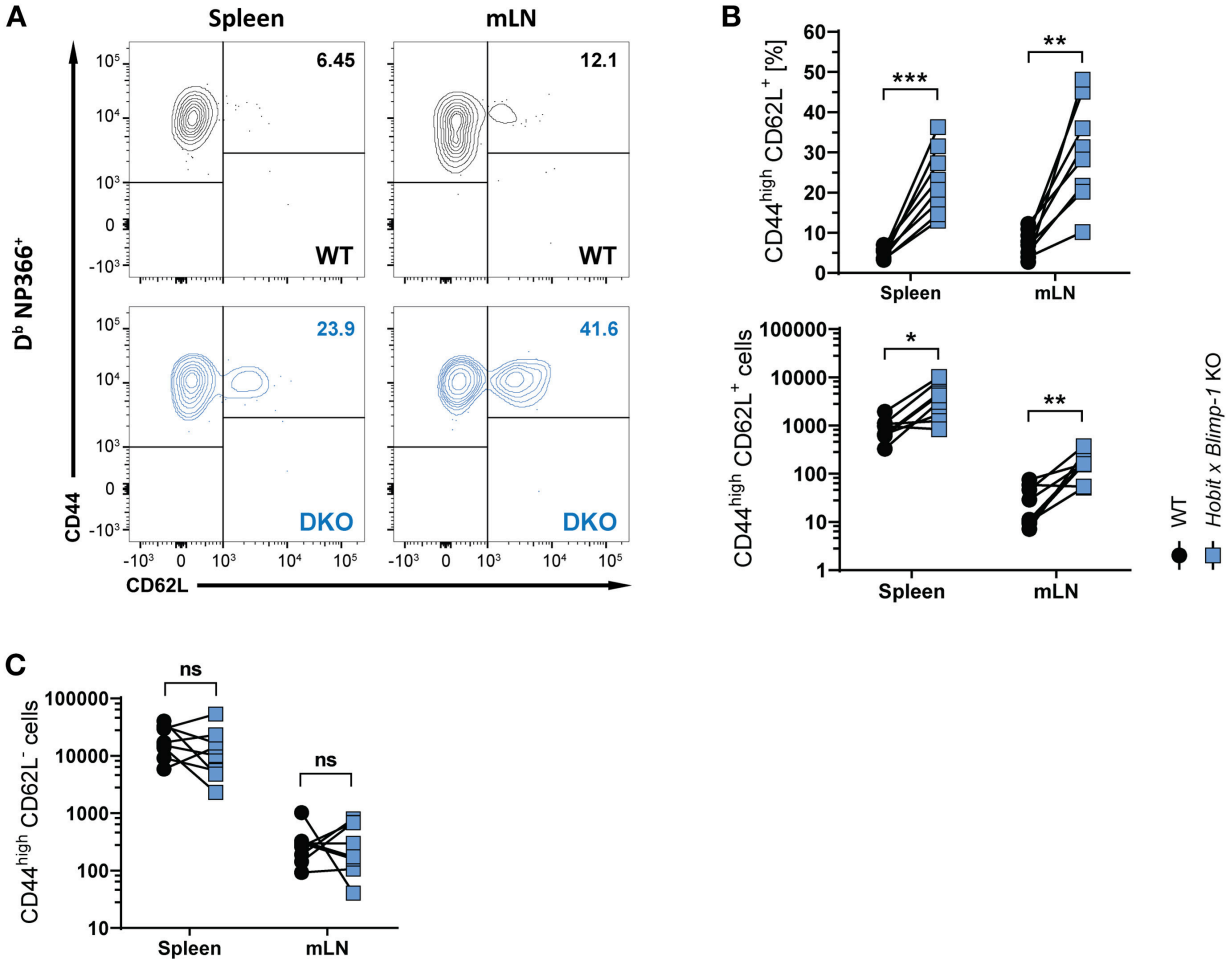
Increased CD8^+^ T_CM_ formation upon combined loss of Hobit and Blimp-1. **(A–C)** WT: Hobit × Blimp-1 KO (DKO) mixed bone marrow chimeras were infected intranasally with HKx31 influenza virus. The phenotype of virus-specific (D^b^ NP366^+^) WT and DKO donor CD8^+^ T cells was analyzed at day 30+ post infection (memory phase). **(A)** Representative flow cytometry plots show expression of CD44 and CD62L on D^b^ NP366^+^ donor CD8^+^ T cells of the WT and DKO compartment from spleen and mediastinal lymph nodes (mLN). **(B**,**C)** The frequencies and absolute numbers of **(B)** CD44^high^CD62L^+^ D^b^ NP366^+^ donor CD8^+^ T cells and **(C)** absolute numbers of CD44^high^CD62L^−^ D^b^ NP366^+^ donor CD8^+^ T cells of the WT and DKO compartment in spleen and mLN were quantified. Combined data from two independent experiments (*n* = 8). Symbols represent individual mice, lines connect paired samples. Paired *t*-test. ^*^P < 0.05; ^**^P < 0.01; ^***^P < 0.001, ns: not significant.

Apart from CD8^+^ T_RM_ cells in the lung, influenza virus infection also induces the formation of circulating effector memory (T_EM_) and central memory (T_CM_) CD8^+^ T cells (30, 31). To assess the impact of co-deficiency of Hobit and Blimp-1 on these circulating memory subsets, we analyzed virus-specific (D^b^ NP366^+^) WT and DKO cells isolated from secondary lymphoid organs of mixed BM chimeric mice after HKx31 infection. In both spleen and lung-draining lymph nodes (mediastinal lymph nodes, mLN), virus-specific CD44^high^ CD62L^+^ CD8^+^ T_CM_ cells were present at elevated levels in the DKO compartment compared to the WT compartment (Figures 3A,B), both in frequencies and in absolute numbers. In contrast, no effect of Blimp-1 and Hobit deficiency was observed in the CD44^high^ CD62L^−^ CD8^+^ T_EM_ subset, as these cells were present in both the WT and the DKO compartment in similar numbers (Figure 3C). Taken together, these data suggest that Hobit and/or Blimp-1 not only drive the formation of CD8^+^ T_RM_ cells, but also suppress the development of CD8^+^ T_CM_ cells after influenza virus infection.

### Generation of Lung CD8^+^ T_RM_ Cells Depends on Blimp-1, but Not Hobit

**Figure 4:**
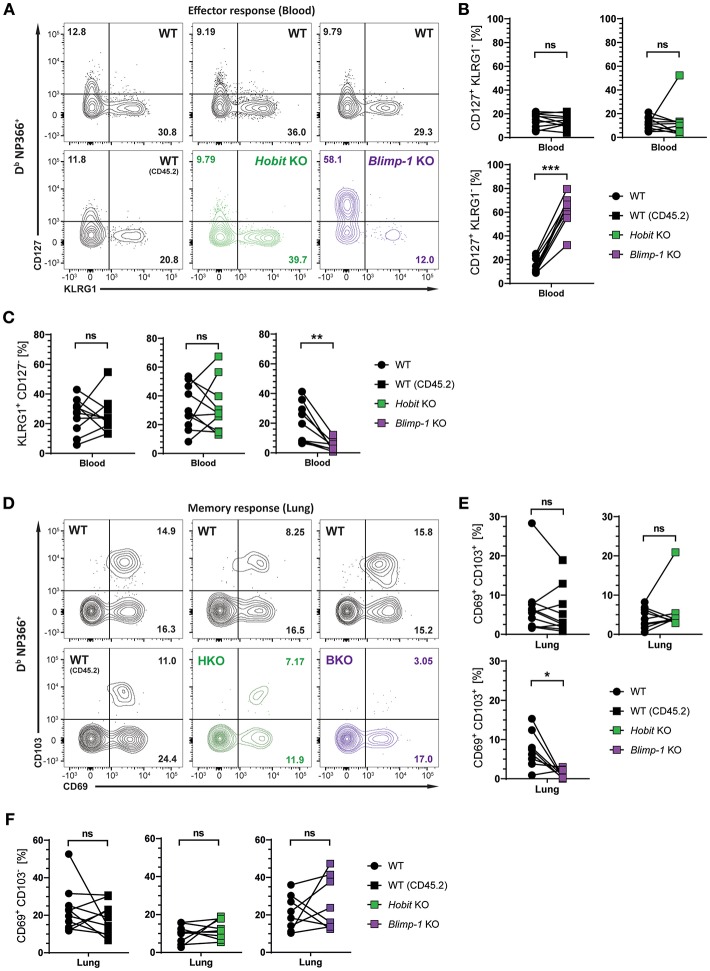
Formation of lung CD8^+^ T_RM_ cells depends on Blimp-1, but not Hobit. Mixed bone marrow chimeras [WT:WT; WT:Hobit KO (HKO); WT:Blimp-1 KO (BKO)] were infected intranasally with HKx31 influenza virus. The phenotype of virus-specific (D^b^ NP366^+^) WT (CD45.1^+^), WT (CD45.2^+^), HKO (CD45.2^+^), and BKO (CD45.2^+^) donor CD8^+^ T cells was analyzed. **(A)** Representative flow cytometry plots show expression of CD127 and KLRG1 by D^b^ NP366^+^ donor CD8^+^ T cells from the blood of mixed bone marrow chimeras at day 10 post infection. **(B**,**C)** Frequencies of **(B)** CD127^+^KLRG1^−^ and **(C)** KLRG1^+^CD127^−^ D^b^ NP366^+^ donor CD8^+^ T cells from the blood of mixed bone marrow chimeras at day 10 post infection were quantified. **(D)** Representative flow cytometry plots show expression of CD69 and CD103 by D^b^ NP366^+^ donor CD8^+^ T cells from the lungs at day 30+ post infection. **(E,F)** Frequencies of **(E)** CD69^+^CD103^+^ and **(F)** CD69^+^CD103^−^ D^b^ NP366^+^ donor CD8^+^ T cells from the lungs at day 30+ post infection were quantified. Combined data from two independent experiments (*n* = 9–10). Symbols represent individual mice, lines connect paired samples. Paired *t*-test. ^*^P < 0.05; ^**^P < 0.01; ^***^P < 0.001, ns: not significant.

We next investigated whether Hobit and Blimp-1 collaborated in the development of lung CD8^+^ T_RM_ cells, as observed in other tissues (20), or whether either one was the dominant transcription factor driving CD8^+^ T_RM_ formation in the lungs. To this end, three groups of mixed BM chimeric mice were generated, consisting of one control group, containing two WT compartments (CD45.1 and CD45.2) and two experimental groups, containing a WT (CD45.1) compartment next to either a Hobit KO (CD45.2) compartment or a Blimp-1 KO (CD45.2) compartment. The mixed BM chimeric mice were infected intranasally with HKx31 virus, and the virus-specific (D^b^ NP366^+^) CD8^+^ T cell response was analyzed over time. As expected, similar to cells lacking both functional Hobit and Blimp-1 (Figures 2C,D), D^b^ NP366^+^ Blimp-1 KO cells contained elevated frequencies of CD127^+^ KLRG1^−^ MPECs at the peak of the anti-viral CD8^+^ T cell effector response (Figures 4A,B). Moreover, D^b^ NP366^+^ Blimp-1 KO cells nearly lacked KLRG1^+^ CD127^−^ TECs, which was also observed for cells with combined deficiency for Hobit and Blimp-1 (Figures 2C,D). Neither of these phenotypes was observed for D^b^ NP366^+^ Hobit-deficient cells, which contained TECs and MPECs at similar frequencies as their WT counterparts (Figures 4A–C). These data indicate a dominant role of Blimp-1 over Hobit in regulating the balance between terminal effector and memory precursor cell differentiation during the effector response. After clearance of the infection, the D^b^ NP366^+^ Hobit KO compartment exhibited no defects in the formation of CD8^+^ T_RM_ cells in the lung (Figures 4D,E). In contrast, the Blimp-1 deficient compartment of virus-specific cells was severely impaired in the formation of CD69^+^ CD103^+^ CD8^+^ T_RM_ cells (Figures 4D,E). As previously observed for cells with combined deficiency for Hobit and Blimp-1, D^b^ NP366^+^ Blimp-1 KO cells did not exhibit a substantial defect in generating CD69^+^ CD103^−^ cells in the lung (Figure 4F). Thus, Blimp-1, but not Hobit, appears to be essential for the formation of terminal effector cells and CD69^+^ CD103^+^ CD8^+^ T_RM_ cells in the lung.

### Blimp-1 Suppresses CD8^+^ T_CM_ Formation

**Figure 5:**
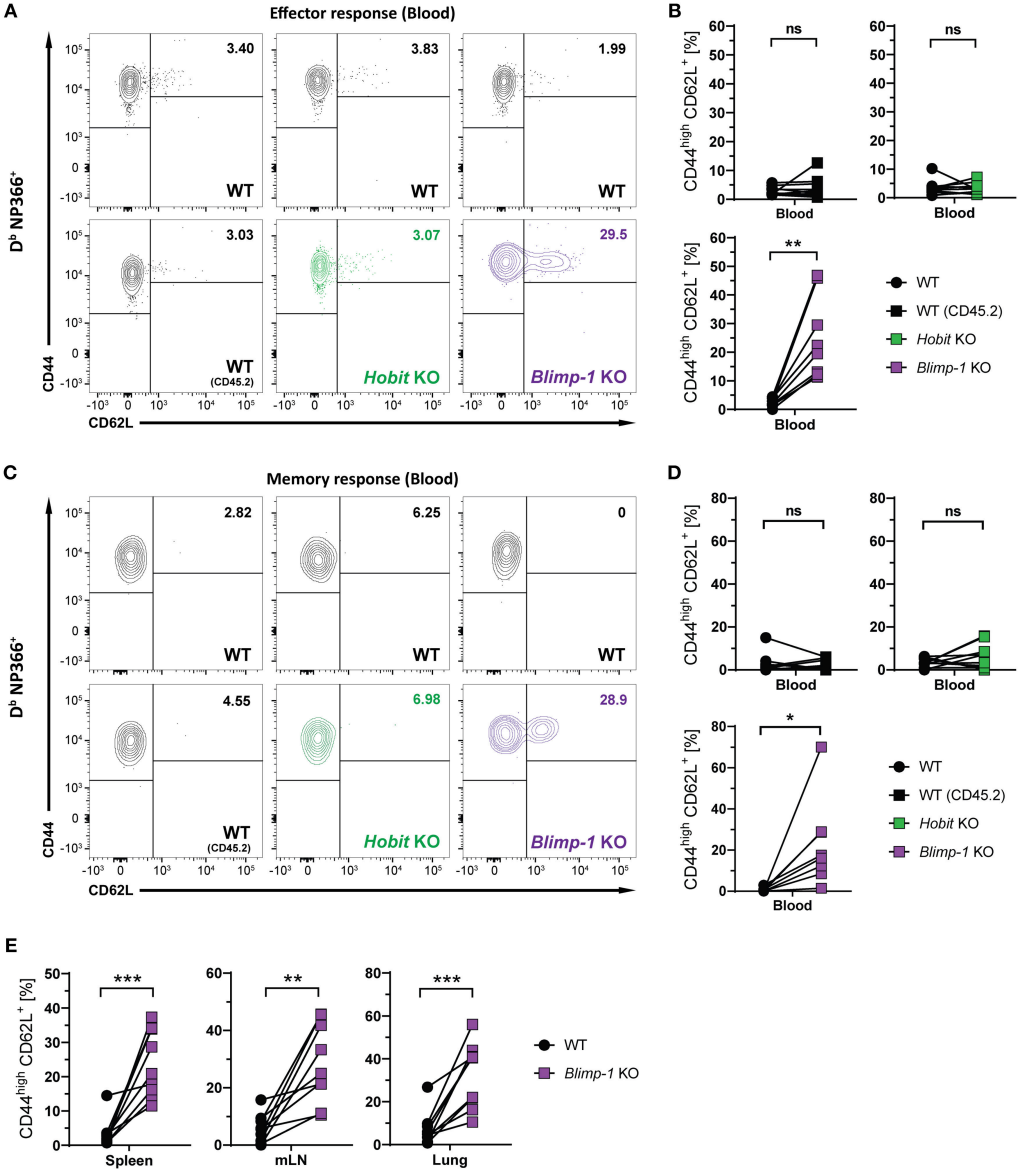
CD8^+^ T_CM_ formation is suppressed by Blimp-1. Mixed bone marrow chimeras [WT:WT; WT:Hobit KO (HKO); WT:Blimp-1 KO (BKO)] were infected intranasally with HKx31 influenza virus. The phenotype of virus-specific (D^b^ NP366^+^) WT (CD45.1^+^), WT (CD45.2^+^), HKO (CD45.2^+^) and BKO (CD45.2^+^) donor CD8^+^ T cells was analyzed in blood **(A–D)** and in the indicated tissues **(E)**. **(A,C)** Representative flow cytometry plots show expression of CD44 and CD62L on D^b^ NP366^+^ donor CD8^+^ T cells from the blood at **(A)** day 10 and **(C)** day 30+ post infection. **(B,D,E)** Frequencies of CD44^high^CD62L^+^ cells D^b^ NP366^+^ donor CD8^+^ T cells from **(B)** blood at day 10 post infection, and from **(D)** blood as well as from **(E)** spleen, mediastinal lymph nodes (mLN) and lung at day 30+ post infection were quantified. Combined data from two independent experiments (*n* = 9–10). Symbols represent individual mice, lines connect paired samples. Paired *t*-test. ^*^P < 0.05; ^**^P < 0.01; ^***^P < 0.001, ns: not significant.

We next considered the role of Blimp-1 and its homolog Hobit in the formation of other memory CD8^+^ T cell subsets. Consistent with previous research demonstrating the repressive role of Blimp-1 in the formation of CD8^+^ T_CM_ cells (24, 25), we observed elevated frequencies of CD44^high^ CD62L^+^ D^b^ NP366^+^ cells in the Blimp-1 KO compartment of mixed BM chimeras already during the effector CD8^+^ T cell response (Figures 5A,B). Importantly, Blimp-1 deficiency resulted in a strong increase in the frequency of D^b^ NP366^+^ CD8^+^ T_CM_ cells in the circulation, lung and secondary lymphoid organs during the memory phase (Figures 5C–E). This effect was not observed for cells lacking functional Hobit, neither during the effector nor the memory response (Figures 5B,D). Consequently, these data suggest that Blimp-1 suppresses CD8^+^ T_CM_ development as early as in the effector response, while Hobit does not appear to have an essential role in this process.

### Blimp-1 Suppresses TCF-1 Expression in Lung CD8^+^ T_RM_ Cells

**Figure 6:**
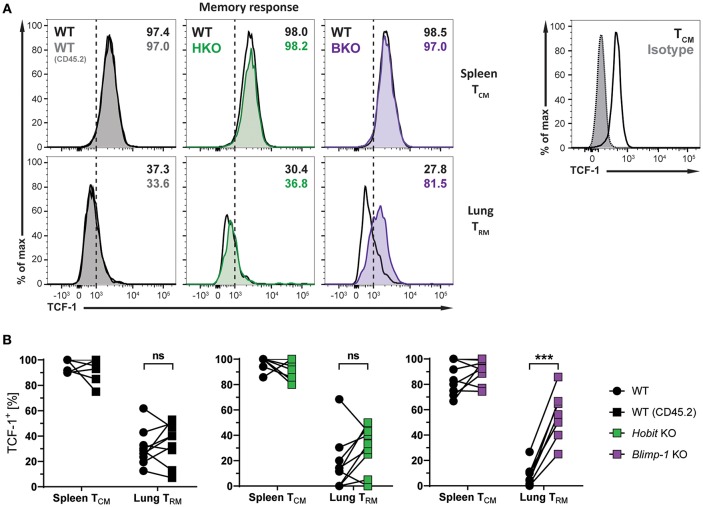
TCF-1 expression in lung CD8^+^ T_RM_ cells is suppressed by Blimp-1. **(A,B)** Mixed bone marrow chimeras [WT:WT; WT:Hobit KO (HKO); WT:Blimp-1 KO (BKO)] were infected intranasally with HKx31 influenza virus. At day 30+ post infection (memory phase), the expression of TCF-1 by virus-specific (D^b^ NP366^+^) WT (CD45.1^+^), WT (CD45.2^+^), HKO (CD45.2^+^), and BKO (CD45.2^+^) donor CD8^+^ T cells was analyzed. Expression of TCF-1 was analyzed in donor CD8^+^ T_CM_ cells (CD44^high^CD62L^+^) from spleen and donor CD8^+^ T_RM_ cells (CD69^+^CD103^+^) from lungs. TCF-1 staining was validated using isotype control staining on CD8^+^ T_CM_ cells (CD44^high^CD62L^+^) from spleen. **(A)** Representative histograms show expression of TCF-1, and **(B)** graphs display frequencies of TCF-1^+^ cells. Combined data from two independent experiments (*n* = 9–10). Symbols represent individual mice, lines connect paired samples. Paired *t*-test. ^***^P < 0.001, ns: not significant.

The transcription factors Hobit and Blimp-1 regulate the development of CD8^+^ T_RM_ cells in part by suppressing genes, which are incompatible with the establishment of tissue-residency (20). A direct target of both Hobit and Blimp-1 is T-cell factor 1 (TCF-1). TCF-1 is a nuclear effector of the canonical Wingless/Integration 1 (Wnt) signaling pathway and constitutes an essential transcription factor for the development of CD8^+^ T_CM_ cells (32, 33). Given the enhanced formation of CD8^+^ T_CM_ cells upon genetic ablation of Blimp-1, we analyzed TCF-1 expression in different memory CD8^+^ T cells subsets arising after influenza infection. As expected, WT D^b^ NP366^+^ CD8^+^ T_CM_ cells exhibited high uniform expression of TCF-1 at the protein level, while virus-specific WT CD8^+^ T_RM_ cells in the lung showed markedly lower expression of TCF-1 (Figures 6A,B). Hobit deficiency did not significantly impact TCF-1 expression in both CD8^+^ T_CM_ and T_RM_ populations. Loss of Blimp-1 had no effect on the high levels of TCF-1 expression in CD8^+^ T_CM_ cells. However, Blimp-1 deficiency resulted in strongly increased TCF-1 protein expression in the remaining virus-specific CD8^+^ T_RM_ cells present in the lung (Figures 6A,B). These results indicate that Blimp-1 mediates suppression of TCF-1 expression in lung CD8^+^ T_RM_ cells, which may contribute to the instruction of CD8^+^ T_RM_ development in the lungs.

## Discussion

The maintenance of lung CD8^+^ T_RM_ cells is distinct from that of CD8^+^ T_RM_ cells at other sites. In contrast to CD8^+^ T_RM_ cells in skin and small intestine, lung-resident CD8^+^ T cells are not maintained as stable populations, but slowly decline over time. Here, we show that CD8^+^ T_RM_ cells in the lungs diverge from T_RM_ cells in the liver, intestine and skin in terms of their transcriptional regulation. Unlike their counterparts in other tissues, differentiation and/or maintenance of lung CD8^+^ T_RM_ cells after influenza virus infection was entirely independent of Hobit. Instead, we found that CD103^+^ lung CD8^+^ T_RM_ cells exclusively depended on Blimp-1. Blimp-1 repressed the formation of virus-specific CD8^+^ T_CM_ cells and the expression of the transcription factor TCF-1 in lung CD8^+^ T_RM_ cells. These findings may suggest that Blimp-1 controls the lineage choice between CD8^+^ T_CM_ and T_RM_ cells after influenza infection and highlight a unique aspect of the transcriptional regulation of CD8^+^ T_RM_ cells in the lung.

In the skin, liver, kidney, and small intestine, CD8^+^ T_RM_ cell formation and/or maintenance is co-regulated by Hobit and Blimp-1 (20). Our data demonstrate that CD103^+^ CD8^+^ T_RM_ cells in the lungs do not require Hobit for their formation, but entirely rely on Blimp-1. These findings may reflect differences in signals from the local microenvironment modulating Hobit and Blimp-1 expression. While influenza virus causes an acute infection of the airways, residual viral antigen can persist in the lung and the draining lymph nodes for extended periods of time after clearance of the infection (34, 35). Presence of remaining viral antigen can induce T cell responses long after the infection and modulates the migration and localization of virus-specific memory CD8^+^ T cells (34–36). Persistent antigen may thus impact on the local CD8^+^ T_RM_ cell pool in the airways. Previous research has demonstrated the requirement of local antigen encounter for the establishment of CD8^+^ T_RM_ cells in the lungs (37). However, it remains unclear whether residual antigen also influences the maintenance of lung CD8^+^ T_RM_ cells. TCR signaling can induce *Blimp-1* expression in T cells (38). Persistent viral antigen in the lungs may thus modulate Blimp-1 expression in CD8^+^ T_RM_ cells at this site and in turn bias the dependence of lung CD8^+^ T_RM_ cells toward Blimp-1. In addition, *Blimp-1* expression is upregulated by pro-inflammatory cytokines, including IL-2 and IL-12 (39). After influenza virus infection, CD8^+^ T_RM_ cells localize to sites of tissue regeneration in the lungs (18), which may be characterized by residual inflammation, thus favoring *Blimp-1* expression. Our transcriptional analysis revealed selective upregulation of the IL-2 receptor alpha chain (*Il2ra*) by CD69^+^ CD8^+^ T_RM_ cells arising in the lungs after influenza infection, potentially indicating increased responsiveness to IL-2. Furthermore, IL-2 signaling is required for residency of CD4^+^ T_RM_ cells in the lungs (40). Consequently, IL-2 and other pro-inflammatory cytokines may also act on lung CD8^+^ T_RM_ cells and enhance Blimp-1 expression, resulting in Blimp-1 dependent maintenance of these memory T cells.

In contrast to Blimp-1, Hobit does not appear to play an essential role in the differentiation and/or maintenance of lung CD8^+^ T_RM_ cells. Nevertheless, Hobit is specifically expressed by CD69^+^ CD8^+^ T cells in the lungs after influenza virus infection, indicating the presence of local cues driving Hobit mRNA expression. The cytokine interleukin-15 (IL-15) mediates *Hobit* expression in a T-bet-dependent manner (20). Local IL-15 and potential other signals may thus promote Hobit mRNA expression in lung CD8^+^ T_RM_ cells. Epithelial cells in the lung airways constitutively express IL-15 (41), and the cytokine mediates the recruitment of effector CD8^+^ T cells into the inflamed lung after influenza virus infection (42). Furthermore, IL-15 is required for persistence of CD103^+^ CD8^+^ T_RM_ cells in the lungs (43), as well as for CD8^+^ T_RM_ maintenance in skin, salivary glands, and kidney (43, 44). Taken together, these findings suggest a prominent role for IL-15 in modulating CD8^+^ T cell responses in the lungs. Whether IL-15 facilitates Hobit expression in lung CD8^+^ T_RM_ cells in a similar fashion as in CD8^+^ T_RM_ cells at other sites, remains to be determined. Despite being differentially expressed, Hobit does not appear to have an essential role in the formation of lung CD8^+^ T_RM_ cells. Using transcriptional profiling, we have detected Hobit and Blimp-1 expression at the mRNA level. However, this does not necessarily reflect the expression of Hobit and Blimp-1 at the protein level, which may be subject to posttranscriptional regulation. Currently, there are no tools available to study Hobit protein expression in mice. Regarding Blimp-1, we have recently demonstrated a discrepancy between the expression of Blimp-1 at the transcriptional and protein level in memory CD8^+^ T cells (9), suggesting regulation at the level of translation or degradation. In line with this, Blimp-1 is similarly expressed by both CD69^+^ and CD69^−^ memory CD8^+^ T cells in the lungs at the transcript level, while Blimp-1 deficiency only perturbs the formation of lung CD8^+^ T_RM_ cells. Therefore, differences at the level of posttranscriptional regulation of Hobit and Blimp-1 expression may shape the unique dependency of lung CD8^+^ T_RM_ cells on Blimp-1.

Our data demonstrate that lung CD8^+^ T_RM_ cells do not require Hobit for their development, and this may have implications for their effector functions. In contrast to circulating memory CD8^+^ T cells, CD8^+^ T_RM_ cells in many tissues maintain high levels of the cytotoxic mediator granzyme B (8, 26, 45). On a transcriptional level, we could show that lung CD8^+^ T_RM_ cells express elevated levels of granzyme B mRNA. However, lung CD8^+^ T_RM_ cells do not retain granzyme B protein expression and exhibit little cytotoxicity (46, 47). We recently showed that Hobit is essential for maintenance of granzyme B expression in liver and intestinal CD8^+^ T_RM_ cells (9). This may indicate a functional link between the poor cytolytic potential of lung CD8^+^ T_RM_ cells and their independence from Hobit-mediated regulation. Whether Blimp-1 and/or Hobit regulate effector responses of lung CD8^+^ T_RM_ cells upon reinfection, remains to be determined.

Immunosurveillance of peripheral tissues for reinfection is primarily performed by CD8^+^ T_RM_ cells (48). These resident populations are characterized by shared phenotypic, transcriptional and functional features, but also exhibit distinct differences across tissues (20, 28). In addition, growing evidence suggests further heterogeneity of CD8^+^ T_RM_ cells within one tissue. Expression of CD103 and CD49a delineates CD8^+^ T_RM_ cell populations with discrete functional capacities in the intestine and skin, respectively (29, 49). In the lung, both CD69^+^ CD103^−^ and CD69^+^ CD103^+^ CD8^+^ T_RM_ cells arise after influenzas virus infection. While genetic ablation of Blimp-1 greatly impaired the formation of CD69^+^ CD103^+^ CD8^+^ T_RM_ cells, the CD69^+^ CD103^−^ population was less affected, suggesting distinct transcriptional regulation of the two subsets. Moreover, CD103^−^ CD8^+^ T_RM_ cells may occupy different sites, as they are unable to undergo interaction with E-cadherin expressing epithelial cells, a process requiring CD103 expression (50, 51). In line with this, CD103^−^ CD8^+^ T_RM_ cells in the intestine display localization and functional properties separate from their CD103^+^ counterparts (49). Hence, lung tissue may harbor distinct CD8^+^ T_RM_ cell populations, delineated by CD103 expression and dependency on Blimp-1.

The transcriptional programs governing the differentiation of naïve CD8^+^ T cells into different subsets of memory CD8^+^ T cells, namely T_CM_, T_EM_ and T_RM_ cells, are gradually being uncovered. However, it is still incompletely understood how transcription factors control the fate choice between the memory CD8^+^ T cell subsets. Previous research has highlighted the importance of the transcription factors Runx3 and Notch in the formation and/or maintenance of CD8^+^ T_RM_ cells in the lungs. While Runx3 is required for the formation of lung CD8^+^ T_RM_ cells, Notch regulates the maintenance of these cells (23, 52). Here, we show that the transcription factor Blimp-1 promotes formation of lung CD8^+^ T_RM_ cells, while suppressing the development of CD8^+^ T_CM_ cells. These phenotypes were confined to the Blimp-1 deficient, but not the WT compartment of mixed BM chimeras, and are thus likely due to cell-intrinsic effects rather than indirect effects involving competition for shared resources. Interestingly, Blimp-1 had no impact on the formation of CD44^high^ CD62L^−^ CD8^+^ T_EM_ cells, indicating that this transcription factor primarily regulates the lineage choice between CD8^+^ T_RM_ and T_CM_ cells. We have previously demonstrated that Blimp-1 suppresses the expression of TCF-1 via direct binding to the TCF-1 encoding *Tcf7* locus. Consistently, *Tcf7* downregulation is a common feature of resident lymphocyte populations (20). We also observed low expression of TCF-1 protein in lung CD8^+^ T_RM_ cells, which was strongly increased upon genetic ablation of Blimp-1. TCF-1 is an essential transcription factor driving the development of circulating memory CD8^+^ T cells, in particular of CD8^+^ T_CM_ cells (32, 33, 53). The suppression of TCF-1 by Blimp-1 may suggest that Blimp-1, in concert with TCF-1, controls the fate choice between CD8^+^ T_RM_ and T_CM_ cells. It will be interesting to determine if and how these two transcription factors drive this lineage choice during CD8^+^ T cell differentiation. This is of particular interest in the context of therapeutic approaches aiming to modulate the balance between different memory CD8^+^ T cell subsets.

Similar to mice, crossprotection against influenza virus in humans is strongly correlated with the presence of CD8^+^ T cells specific to conserved viral epitopes (54, 55). Moreover, CD8^+^ T cells recognizing respiratory viruses, including influenza virus, are enriched in the human lung (56, 57). A substantial fraction of these virus-specific CD8^+^ T cells express markers of tissue-residency, including CD69 and CD103 (58, 59), indicating the presence of bona fide influenza-specific CD8^+^ T_RM_ cells within the human lung. Transcriptional profiling of human lung CD8^+^ T_RM_ cells has demonstrated a strong overlap with gene signatures of CD8^+^ T_RM_ cells from different murine tissues, including the selective upregulation of tissue retention molecules (e.g., CD49a, CD103, CD69), and the downregulation of tissue egress receptors (e.g., S1PR1, S1PR5) (23, 28). In line with our findings in mice, CD8^+^ T_RM_ cells in the human lung exhibit no differential expression of Blimp-1 on the transcriptional level in comparison to circulating CD8^+^ T_EM_ cells (23). In contrast to their murine counterparts, Hobit mRNA is not differentially expressed in human lung CD8^+^ T_RM_ cells, relative to the expression in CD8^+^ T_EM_ cells (23, 28), partly because the latter population also expresses Hobit in humans (23, 60). This may suggest that Hobit is not specifically involved in the differentiation of human lung CD8^+^ T_RM_ cells. It remains to be determined if Blimp-1, on the other hand, promotes CD8^+^ T_RM_ differentiation in humans.

We show that CD103^+^ CD8^+^ T_RM_ cell formation in the lungs after influenza virus infection is dependent on the transcription factor Blimp-1. Blimp-1 driven suppression of TCF-1 expression may instruct the lineage choice between CD8^+^ T_CM_ and T_RM_ cells during memory CD8^+^ T cell formation. As there is growing recognition of the clinical importance of tissue-resident T cell memory, we believe that insights into the transcriptional mechanisms governing local CD8^+^ T_RM_ cell differentiation are an important prerequisite for the development of novel vaccination approaches in the lungs.

## Materials and Methods

### Mice

C57BL/6JRj (CD45.2^+^ WT) mice were purchased from Janvier and B6.SJL-*Ptprc*^*a*^ *Pepc*^*b*^/BoyJ (CD45.1^+^ WT) mice from the Jackson Laboratory. Both lines were crossed to generate B6.SJL-*Ptprc*^*a*^ *Pepc*^*b*^/BoyJ x C57BL/6JRj mice. *Zfp683*^−/−^ (*Hobit* KO) (61), *Prdm1*^flox/flox^ × Lck Cre (*Blimp-1* KO) mice (24) and *Zfp683*^−/−^ × *Prdm1*^flox/flox^ × Lck Cre mice (20) were maintained on a C57BL/6JRj background. For the generation of mixed bone marrow (BM) chimeras, CD45.1 × CD45.2 (B6.SJL-*Ptprc*^*a*^ *Pepc*^*b*^/BoyJ × C57BL/6JRj) recipient mice were irradiated (2x 5 Gy) and reconstituted with i.v. transfer of 10^*^10^6^ BM cells per genotype. Mixed BM chimeras were used 12–16 weeks after reconstitution and chimerism of lymphocytes was confirmed prior to usage. All mice were maintained under SPF conditions in the animal facility of the Netherlands Cancer Institute (NKI). Animal experiments were conducted according to institutional and national guidelines.

### Influenza Virus Infection

Mice were infected intranasally with 100x TCID_50_ (median tissue culture infectious dose) of HKx31 influenza A virus in a volume of 50 μl after anesthetization by inhalation of isoflurane. HKx31 virus was kindly provided by Dr. Guus Rimmelzwaan (University of Veterinary Medicine Hannover). At the indicated time points after infection, mice were sacrificed, and tissues were harvested for analysis of CD8^+^ T cell responses.

### Tissue Preparation

Single cell suspensions from spleen and lymph nodes were prepared by mechanical disruption via passing of the tissues over a 70 μm cell strainer. Lung tissue was cut into pieces of 1 mm^2^ and enzymatically digested for 30 min at 37°C with 750 U ml^−1^ Collagenase Type I (Worthington) and 0.31 mg ml^−1^ DNase I (Roche, from bovine pancreas, grade II) in RPMI 1,640 supplemented with 10% (v/v) fetal calf serum (FCS). Single cell suspensions were generated by filtering over a 70 μm cell strainer and the isolated lymphocytes were purified by density centrifugation on a 66/44% Percoll gradient (GE Healthcare). Lung lymphocytes were extracted from the interphase of the Percoll gradient. Contaminating erythrocytes were removed using red blood cell lysis buffer (155 mM NH_4_Cl, 10 mM KHCO_3_, 0.1 mM EDTA).

### Flow Cytometry

Cells were incubated with antibodies and tetramers for 25 min at 4°C and washed with PBS supplemented with 0.5% (v/v) FCS. The following anti-mouse monoclonal antibodies were used: anti-CD3 (17A2), anti-CD4 (RM4-5), anti-CD8a (53–6.7), anti-CD44 (IM7), anti-CD45.1 (A20), anti-CD45.2 (104), anti-CD49a (Ha31/8), anti-CD62L (MEL-14), anti-CD69 (H1.2F3), anti-CD103 (M290), anti-CD127 (A7R34), anti-CXCR6 (SA051D1), anti-KLRG1 (2F1), anti-TCF-1 (S33-966), mouse IgG1, κ isotype control (P3.6.2.8.1). Antibodies were purchased from BioLegend, eBiosciences, BD Biosciences, or BD Pharmingen. Influenza-virus-specific CD8^+^ T cells were detected using H-2 D^b^ ASNENMETM (NP_366−374_) tetramers (D^b^ NP366), which were a kind gift of Dr. Anja ten Brinke (Sanquin Research). Exclusion of dead cells was performed with live/dead fixable near-IR dead cell stain kit (Thermo Fisher Scientific). For staining of intracellular molecules, the Foxp3/Transcription Factor Staining Buffer Set (eBioscience) was used according to the manufacturer's specifications. Samples were acquired on an LSR Fortessa flow cytometer (BD) and data was analyzed using FlowJo V10 (Tree star) software.

### RNA-Seq Analysis

A subset of previously published RNA-seq (CEL-seq) data (geo accession number GSE79774) (23) was reanalysed. DMSO-treated CD69^+^ and CD69^−^ memory (CD44^high^ CD62L^lo^) CD8^+^ T cells isolated from murine lungs at day 30+ after HKx31 infection were analyzed to determine the tissue-resident phenotype of these populations. Reads were first aligned to mm10 genome with STAR, followed by read count quantification with featureCounts using Ensembl's v92 annotation with modification of Z*fp683*. Since CEL-seq was used, only the 3′ end of the genes is sequenced, and inspecting the alignments manually revealed that the reads that mapped to *Zfp683* were outside Ensembl's defined region of the gene. We therefore extended the 3′ end of *Zfp683* with 1,057 nucleotides to include all reads of this gene. To find differentially expressed (DE) genes, first low expressed genes were removed (retained genes had more than 1 cpm (counts per million) in at least four samples), followed by limma voom and quantile normalization. A linear model was fitted to each gene using a design that also included mouse (paired) and library (batch) effect, and eBayes moderated t-statistics were used to find DE genes between CD69^+^ and CD69^−^ memory CD8^+^ T cells. Benjamini-Hochberg procedure was used for FDR correction, and adjusted *P* < 0.05 were considered significant.

### Statistical Analysis

For pairwise comparisons, a standard two-sided Student's *t*-test (paired), was applied with GraphPad Prism 6 software. *P* < 0.05 were considered statistically significant (^*^P < 0.05; ^**^P < 0.01; ^***^P < 0.001). The Morpheus software (https://software.broadinstitute.org/morpheus), developed by the Broad Institute, was used to generate heat maps. Values were converted to heat map colors using the mean and maximum values for each row.

## Ethics Statement

This study was carried out in accordance with the national guidelines of the Central Commission for Animal Experiments (Centrale Commissie Dierproeven). The experimental protocols were approved by the Animal Welfare Body of the Netherlands Cancer Institute (NKI).

## Author Contributions

FB and KvG conceived and designed studies. FB, NK, TW, and RS performed animal experiments and FB analyzed data. BN analyzed RNAseq data provided by PH and DA. FB and KvG wrote the manuscript. All authors provided critical feedback and approved the manuscript.

### Conflict of Interest Statement

The authors declare that the research was conducted in the absence of any commercial or financial relationships that could be construed as a potential conflict of interest.
